# Effects of Lymphaticovenous Anastomosis on Quality of Life, Body Image, and Spiritual Health in Lymphedema Patients: A Prospective Cohort Study

**DOI:** 10.3390/healthcare12141419

**Published:** 2024-07-16

**Authors:** Shu-Hui Peng, Ching-Ya Huang, Chun-Ming Shih, Pei-Yu Tsai, Johnson Chia-Shen Yang, Ching-Hua Hsieh

**Affiliations:** 1 Department of Plastic Surgery, Kaohsiung Chang Gung Memorial Hospital, Chang Gung University College of Medicine, Kaohsiung 83301, Taiwan; pshui@cgmh.org.tw (S.-H.P.); b101106030@tmu.edu.tw (C.-Y.H.); mermaid85@cgmh.org.tw (P.-Y.T.); 2 Graduate School of Human Sexuality, Shu-Te University, Kaohsiung 824445, Taiwan; cmshih0901@stu.edu.tw

**Keywords:** lymphaticovenous anastomosis (LVA), lymphedema, quality of life (QOL), body image, spiritual health

## Abstract

Background: Lymphedema is a debilitating condition that significantly affects quality of life due to its chronic nature and visible symptoms. Lymphaticovenous anastomosis (LVA) has emerged as a promising surgical intervention, yet its effects on body image and spiritual health alongside physical symptoms have not been thoroughly examined. This study evaluates the efficacy of LVA in improving symptoms, quality of life (QOL), body image, and spiritual well-being in lymphedema patients. Methods: A prospective cohort study was conducted at Kaohsiung Chang Gung Memorial Hospital, Taiwan, involving 44 patients with lymphedema undergoing LVA surgery. Evaluations were made pre-surgery, one month post-surgery, and six months post-surgery using the 36-Item Short Form Health Survey (SF-36), Multidimensional Body–Self Relations Questionnaire-Appearance Scales (MBSRQ-AS), and a spiritual health scale. Statistical analysis was performed using one-way repeated measures ANOVA. Results: Significant improvements were observed in lymphedema symptoms and QOL measures at six months post-operation. SF-36 results showed enhanced scores in nearly all domains, particularly in physical functioning and role-physical. The appearance orientation scores from the MBSRQ-AS significantly increased, indicating improved perceptions in some dimensions of body image. Conclusions: LVA surgery significantly enhances physical and psychological outcomes in patients with lymphedema, with marked improvements in symptoms, QOL, and body image perceptions. The findings suggest that while LVA is effective in addressing the physical and psychological aspects of lymphedema, it does not impact spiritual dimensions. This underscores the need for holistic approaches in the management of lymphedema to address all facets of patient well-being.

## 1. Introduction

Lymphedema is defined as an external and/or internal manifestation of lymphatic system insufficiency and deranged lymph transport. It is considered a symptom or sign resulting from underlying lymphatic disease and is recognized as an illness by the World Health Organization [1]. Primary lymphedema arises from inherent malformations or dysfunctions of the lymphatic system and can manifest at various stages of life, often exacerbating during puberty or pregnancy [2,3]. Secondary lymphedema is more prevalent and frequently results from oncological treatments such as lymph node dissection and radiation therapy, particularly in breast cancer patients [4]. Obesity, procedures like surgery, or conditions such as bacterial infection can also lead to lymphatic drainage impairment [5,6,7] and contribute to the development of lymphedema [5,7,8]. This condition can lead to a range of complications, including recurrent infections, fibrosis, and severe disfigurement, which significantly impair the patient’s quality of life [9].

Lymphedema can have a substantial influence on people’s quality of life (QOL) by impairing physical function, mental health, and social well-being. Physically, patients suffer from swelling, discomfort, and a heavy feeling in the affected limb, which can severely limit mobility and daily activities [4,10,11]. Psychologically, the visible changes in appearance and the chronic nature of the disease can lead to anxiety, depression, and social withdrawal [11,12]. Additionally, the need for ongoing management and therapy can create a significant economic burden, affecting both personal finances and healthcare systems [5,13]. Body image, which refers to an individual’s perceptions, thoughts, and feelings about their physical appearance, is particularly relevant in lymphedema due to the visible changes in affected limbs [11,14]. Alterations in body image can have profound psychological effects, influencing self-esteem, social interactions, and overall well-being [11,14]. Understanding how LVA impacts body image could provide valuable insights into the holistic benefits of this surgical intervention. Spiritual health, encompassing aspects of purpose, meaning, and connectedness, is an increasingly recognized component of overall well-being [15,16]. For individuals with chronic conditions like lymphedema, spiritual well-being can play a crucial role in coping and resilience. However, the impact of lymphedema and its treatments on spiritual health remains largely unexplored.

Current management strategies for lymphedema focus on symptom control and prevention of progression. Manual lymphatic drainage [16], which involves massaging to encourage lymphatic fluid flow and avoid reaccumulation, is used to treat lymphedema in its early stages, whereas compression therapy, which uses elastic bandages or compression clothing, aids lymphatic fluid propulsion [17]. However, such lymphedema treatments frequently have minimal efficacy in the advanced stages and are often palliative rather than curative and require lifelong adherence. Recently, supramicrosurgery, such as lymphaticovenous anastomosis (LVA), has been proved to considerably reduce lymphedema and improve subjective symptoms [18,19,20]. LVA has been utilized to redirect lymphatic flow with functional cutoff sizes greater than 0.5 mm to vein circulation [21]. Patients frequently experience significant alleviation in limb swelling following anastomosis, with objective reductions in limb circumference recorded during follow-up appointments. Patients report tissue softening, weight loss, and a reduction in general discomfort after surgery [20,22,23,24,25,26]. Significant improvements were noted in patient-reported outcomes including pain, heaviness, anxiety, and impact on daily activities such as hobbies, work, and intimacy [27]. Thomas et al. [27] demonstrated a substantial decrease in the need for compression garments and a significant reduction in cellulitis episodes and associated healthcare costs. These findings suggest that LVA effectively halts the progression of lymphoedema and enhances patient well-being.

While lymphedema has a significant impact on the patients’ QOL [10,14,28,29,30,31], and treatment for lymphedema may enhance QOL [10,28,29,30,31], the effect of LVA on QOL was less unexplored. More importantly, there is no literature that investigates the body image and spiritual health of lymphedema patients, or whether lymphedema treatment may significantly enhance how patients perceive changes in their appearance and spiritual well-being. As a result, the purpose of this study is to investigate the changes in QOL, body image, and spiritual health among lymphedema patients undergoing LVA surgery.

## 2. Materials and Methods

### 2.1. Study Designs and Participants

This prospective cohort study was carried out at Kaohsiung Chang Gung Memorial Hospital in Taiwan from April 2019 to December 2020. The study’s sample size was determined using G-Power 3.1, based on a medium effect size (f2 = 0.35), a significance level (alpha) of 0.05, and a power (beta) value of 0.2. To obtain statistical significance, a sample size of 44 people was necessary. Patients with lymphedema who are scheduled to have LVA were included in the study. The patient must be aware and able to communicate before agreeing to participate in the study by signing informed permission. Exclusion criteria included patients with chronic heart failure and renal failure, those who were anticipated to be unable to attend follow-up appointments or complete questionnaires, and those who expressed doubts about the trial. Before beginning the trial, the Chang Gung Memorial Hospital Institutional Review Board (IRB procedure number: 201900305B0) granted ethical approval, and patient consent was obtained as required. All patient consents were acquired by one trained clinical assistant. Purposive sampling and integrated questionnaire surveys were used in this study to investigate body image, QOL, and spiritual health among patients undergoing microsurgical LVA at our institution at three intervals: before surgery (pre-op), one month after surgery (post-op 1 month), and six months after surgery (post-op 6 months). The demographics of the patients were collected, including age, gender, weight, occupation, marital status, education level, monthly economic income, beliefs, and clinical characteristics of lymphedema, such as lymphedema location and limb circumference. The limb circumference was measured according to the procedure described in our prior study [19]. The clinical assistant who did the measurement of the questionnaire is blinded to the results from the measurement of limb circumference.

### 2.2. Study Tools

#### 2.2.1. The Questionnaire for Lymphedema Symptoms

The lymphedema symptom questionnaire evaluates the severity of discomfort caused by nine specific symptoms of lymphedema (1. swelling, 2. heaviness, 3. pain, 4. numbness, 5. impact on appearance change, 6. joint flexibility, 7. walking, 8. climbing stairs, 9. exercise, playing sports, dancing) and the impact on daily activities of three items (1. daily activities, 2. social activities, 3. outdoor activities). The responses are rated on a scale of 0 to 10, where 0 represents normal and 10 represents extremely uncomfortable.

#### 2.2.2. The 36-Item Short Form Health Survey Quality of Life Scale (SF-36), Taiwan Version

This study utilized the 36-Item Short Form Health Survey Quality of Life Scale (SF-36), a multidimensional health-related questionnaire for life quality that was established in 1990 [32]. The Taiwan version (Short Form-36 Taiwan version) [33] was translated and authorized for academic use by domestic scholars following the International Quality of Life Assessment Project. It is a general psychological measurement tool not specifically designed for any particular age group, disease, or treatment. The survey predominantly evaluates the perceived health status of participants along eight dimensions, incorporating a grand total of 36 items. Within these dimensions, there are ten items measuring physical functioning, four measuring role limitations due to physical health problems, two measuring bodily pain, five measuring general health perceptions, four measuring vitality, two measuring social functioning, three measuring role limitations due to emotional health problems, five measuring mental health, and an extra item measuring self-rated health change. In addition, the physical component scale and the mental component scale can be created from the sum of the scores on the first four dimensions and the last four dimensions, respectively. This allows for the separation of the physiological and psychological components of health. The scoring ranges from 0 to 100, where 0 signifies the most deplorable state of health and 100 represents the most favorable state of health.

#### 2.2.3. Multidimensional Body–Self Relations Questionnaire-Appearance Scales (MBSRQ-AS)

The Multidimensional Body–Self Relations Questionnaire-Appearance Scales (MBSRQ-AS) is a questionnaire for self-report that has been purposefully developed to assess dimensions of body image that are associated with physical appearance [34]. The instrument consists of 34 items, which are categorized into four subscales. The response options for items 1 through 22 are outlined on a five-point Likert scale: one (strongly disagree) and five (strongly concur). Evaluating sentiments of physical attractiveness or unattractiveness, as well as contentment or discontentment with one’s appearance. The majority of high scorers are optimistic and content with their physical appearance. This metric measures the degree to which an individual is invested in his or her physical appearance.

Individuals that achieve high scores prioritize their physical appearance, devote attention to their looks, and participate in rigorous grooming practices. The body region satisfaction scale measures the level of satisfaction individuals have with various portions of their body, using a scoring system that ranges from 1 (indicating a high level of dissatisfaction) to 5 (indicating a high level of satisfaction). The overweight preoccupation evaluates a concept that represents excessive concern about fat anxiety, weight vigilance, dieting, and eating restraint. The self-classified weight scale measures an individual’s perception and categorization of their weight, ranging from very underweight to very overweight. It assigns a numerical value of 1 (very underweight) to 5 (very overweight) to indicate the level of weight classification. To calculate scores, the mean of each scale is calculated by inverting the results of six particular categories (namely 11, 14, 16, 18, 19, and 20). The Chinese version had been created with the Cronbach’s α values for these dimensions which are 0.72, 0.84, 0.75, 0.71, and 0.91, respectively [35].

#### 2.2.4. Spiritual Health Scale

The study assessed the spiritual health using the “spiritual health scale” which was originally developed by Howden in 1992 [36] and revised by Fisher, Francis, and Johnson in 2000 [37,38]. The scale comprises five dimensions, with a combined total of twenty-five items: transcending adversity (5), interpersonal relationships (5), religious trust (5), meaning of life (5), and contemplating nature (5). The responses are evaluated using a Likert scale consisting of five points. The scale spans from “strongly disagree” to “strongly agree,” and the relevant scores are 1 to 5. Greater scores on each dimension indicate superior total spiritual well-being, whilst lower values imply worse spiritual well-being.

### 2.3. Statistical Analysis

We coded data from legitimate surveys and analyzed them using the SPSS 22.0 software suite. We utilized one-way repeated measurement ANOVA to evaluate differences between continuous variables within various categories. Depending on the data’s homogeneity, we used Tukey’s HSD post-hoc tests for correction to assess differences in continuous variables between groups. Continuous data were expressed as mean ± standard deviation (SD), whereas categorical variables were expressed as number (percentage). *p*-values < 0.05 were considered statistically significant.

## 3. Results

### 3.1. Patients Demographics

The study included a total of 44 participants who were diagnosed with lymphedema and underwent LVA (Table 1). Out of the patients included in the study, 3 (6.8%) were male and 41 (93.2%) were female, with an average age of 59.30 ± 10.50 years. In total, 72.70% of the individuals were married. The study subjects were primarily patients who had received education at the elementary level (29.5%) and junior high school (20.5%). In terms of religious beliefs, the majority of individuals described themselves as Taoists, accounting for 40.9% of the population, followed by Buddhists, making up 31.8%. In relation to the lymphedema status (Table 2), the majority of patients received the diagnosis within one year (34.1%), while a smaller proportion experienced this condition for a longer duration, with 11.4% having it for 5−10 years, and 18.2% for more than 10 years. 77.3% of the patients exhibited lymphedema at stage II-III, whereas 9.1% had stage I/II, and 4.5% had stage III. The most frequently reported location for lymphedema is the right leg (52.3%), followed by the left leg (38.6%), left arm (6.8%), and right arm (2.3%). Prior to the surgery, the leg had a limb circumference of 377.8 ± 70.5 mm, whereas the arm had a circumference of 226.2 ± 15.4 mm. The limb circumference of the leg and arm one month after the procedure measured 358.1 ± 43.7 and 220 ± 15.9 (mm), respectively. The limb circumference of the leg and arm, measured six months post-operation, was 349.8 ± 46.2 mm and 224.8 ± 13.8 mm, respectively.

### 3.2. Lymphedema Symptoms

Table 3 highlights the significant improvements in lymphedema symptoms observed after the surgery. While heaviness, pain, and numbness showed notable relief as early as one month post-operation, other debilitating symptoms such as swelling, appearance impact, joint flexibility, and mobility impairments demonstrated substantial improvement only at the six-month mark when compared to pre-operative levels. These findings underscore the progressive nature of symptom alleviation following LVA, culminating in significant enhancements by the sixth month post-surgery.

### 3.3. Quality of Life

Table 4 elucidates the surgery’s profound impact on patients’ QOL. The SF-36 QOL assessment revealed substantial improvements across most domains, including physical functioning, role limitations, bodily pain, general health, vitality, and social functioning, at both one and six months post-operatively. Only the domain of mental health did not show significant improvement after the surgery. Notably, the physical and mental component summary scores exhibited a remarkable upward trajectory, peaking at six months post-surgery.

### 3.4. Body Image and Spiritual Health

The MBSRQ-AS body image evaluation indicated a significant increase in appearance orientation scores following LVA (Table 5), implying patients placed greater emphasis on their appearance and engaged in more grooming behaviors post-surgery. While the other four domains, including appearance evaluation, body area satisfactions, overweight preoccupation, and self-classified weight, did not show significant difference after the surgery. Of note, while LVA surgery yielded tangible benefits in symptom relief, QOL, and some body image perceptions, Table 6 reveals no substantial changes in patients’ spiritual well-being scores across all time points. This absence of impact on spiritual health suggests that while the surgery effectively addresses physical and psychological aspects, it may not directly influence an individual’s spiritual outlook or beliefs.

### 3.5. Summary of Results

Collectively, the results of this study paint a comprehensive picture of LVA surgery’s therapeutic potential for lymphedema patients. The data illustrates a progressive improvement in symptoms, QOL, and body image perceptions over time, culminating in substantial enhancements six months post-operatively. However, the lack of change in spiritual well-being scores indicates that the surgery’s benefits are primarily focused on alleviating physical and psychological burdens, leaving spiritual dimensions relatively unaffected.

## 4. Discussion

LVA surgery is widely acknowledged for its ability to treat the debilitating symptoms of lymphedema, such as swelling, discomfort, and restricted mobility. Imai et al. [39] present a compelling case study of LVA-treated lymphedema, showcasing subjective and objective symptom amelioration, reduced swelling, and pain following the operation. Similarly, Boccardo et al. [40] show that LVA is effective in avoiding and treating lymphedema after lymph node dissection in melanoma patients. Their retrospective evaluation revealed a considerable reduction in lymphedema symptoms, with an average 80% reduction in pre-operative excess volume. Oshima et al. [41] underline the importance of early diagnosis and the use of preventive LVA in improving lymphedema prognosis. Their findings indicate that early management can dramatically improve outcomes for people at risk of developing lymphedema, emphasizing the importance of early and accurate identification of high-risk individuals.

In addition to symptom relief, the effect of LVA surgery on lymphedema patients’ QOL has aroused curiosity. Thomas et al. [42] conducted a qualitative study to investigate the impact of LVA on people’s perceptions of their QOL and found significant improvements in symptoms and daily living activities after surgery. Participants reported less anxiety and concern about getting cellulitis, less pain, aching, heaviness, and stiffness, and reduced reliance on compression garments, indicating a return to a more normal lifestyle. Tang et al. [43] performed a systematic assessment of health-related quality of life (HRQOL) outcomes following surgical lymphedema therapy, which supported these findings. Improvements in HRQOL remain for at least 6 to 12 months following surgery. These studies likewise demonstrate the favorable influence of surgical procedures on patients’ HRQOL, which is consistent with our findings. The study’s findings highlight the tremendous impact of surgical intervention on lymphedema patients, with significant symptom relief and QOL improvements over time, particularly six months after surgery. Early postoperative improvements in heaviness, discomfort, and numbness highlight the immediate benefits of surgery, with further functional gains evident as time passes. There was also a consistent improvement in QOL following surgery. These findings are in line with previous research, which has highlighted the positive effects of lymphedema management strategies on patient well-being [18,39,40,42,43,44,45,46], emphasizing the importance of early and effective surgical therapies in the lymphedema therapeutic landscape.

Lymphedema patients may worry about how they look and how visible their condition is, which can have a big effect on how they choose to present themselves [47]. Jager et al. [14] studied women with lymphedema and those who had been injured and discovered that female lymphedema patients possess multiple issues with how they feel about their bodies. Patients may become more aware of and concerned about how they look, which may make them do more things to manage their appearance than what their doctors suggest. This can include changing what they wear to hide swollen areas or an affected limb, as well as changing what they do and how they act in social situations to manage perceptions of their appearance. In this study, we discovered a substantial increase in the appearance orientation score on the MBSRQ-AS assessment, but no significant difference in the other four domains, despite an encouraging trend in appearance evaluation and body area satisfaction following surgery. While both appearance evaluation and appearance orientation are related to body image, they are not the same construct. Appearance evaluation focuses on the self-perceptions and attitudes related to one’s appearance, while appearance orientation focuses on the investment and importance placed on one’s appearance. Appearance orientation refers to the investment in one’s appearance, including the effort and importance placed on being “good-looking” [48]. Conversely, appearance orientation and appearance evaluation are two discrete elements of body image assessment. Self-perceptions and attitudes regarding one’s physical appearance, including attractiveness, thinness, and muscularity, are referred to as appearance evaluation [48,49]. Emotional and psychosocial distress linked to the conditions may be mitigated as a result of the symptomatic alleviation following LVA surgery; this may also promote a more positive body image and self-esteem [39].

The mental load of lymphedema includes feeling left out by healthcare professionals, having problems with body image, losing the ability to do things, and always not knowing what will happen. All of these things add up to a feeling of frustration and anger that builds over time [16,50,51]. Tobin et al. [47] recruited 15 female patients with breast cancer-related arm swelling and showed greater psychological morbidity at the formal psychiatric interview and impaired adjustment to illness as evaluated by the Psychosocial Adjustment to Illness Scale, with the morbidity being particularly in the areas of anxiety and depression. The systematic review [52] emphasizes that distress and anxiety are prevalent among women suffering from breast cancer-related lymphedema. The review identifies that conservative rehabilitation interventions can have varying effects on mental health. For example, face-to-face education programs were found to be more effective than virtual education or social network-based training in improving emotional well-being. Some studies reported that the associated reductions in stress and psychological discomfort in lymphedema treatment could indirectly benefit spiritual health by improving overall well-being [53]. The psychosocial impact of lymphedema includes anxiety, frustration, sadness, and fear, which can significantly affect a patient’s emotional state. Treatments that alleviate these symptoms can indirectly foster a better spiritual environment by reducing emotional distress and enhancing coping capabilities [54]. However, in this study, we did not disclose a significant improvement in spiritual health after LVA surgery. Lymphedema can impose stressful physical and emotional symptoms alongside challenging self-care demands [51]. However, a longer follow-up with multidimensional evaluations may be required for affirmative conclusions.

The study was constrained by several limitations. At first, the study was conducted at a single facility with a limited number of participants, which undermined the relevance of the findings. The limited patient number hindered the later examination of subgroups with diverse backgrounds among the individuals. Furthermore, the duration of the follow-up period was somewhat short, which impeded our ability to evaluate the long-term effects on patients’ outcomes. Moreover, the study’s dependence on self-reported measures to assess body image and spiritual health may introduce bias. Finally, one potential limitation is the lack of stratification by stages of lymphedema and the affected limb location (upper or lower). This oversight may introduce bias, as the severity and impact of lymphedema can vary significantly depending on these factors. In order to address these limitations and gain a more comprehensive understanding of the benefits of LVA surgery for lymphedema patients, incorporating more diverse scales and involving occupational therapists more fully in treatment plans may improve patients’ overall well-being and quality of life. Furthermore, future research should use multi-center designs, larger sample sizes, longer follow-up durations, and objective measurements.

## 5. Conclusions

In conclusion, the study effectively demonstrated that Lymphaticovenous Anastomosis (LVA) surgery provides substantial relief from the physical and psychological burdens of lymphedema. Patients reported significant improvements in symptoms, QOL, and body image perception, particularly six months post-operation. These enhancements contribute profoundly to better overall patient health and daily functioning, despite the unchanged scores in spiritual well-being. The enduring positive outcomes emphasized by the data highlight the pivotal role of LVA in managing lymphedema, underlining its value as a critical intervention for patients suffering from this debilitating condition.

## Figures and Tables

**Table 1:** Demographics of the lymphedema patients undergoing lymphaticovenous anastomosis surgery.

Characteristics		*n* (%)/Mean ± SD
Age, years		59.3 ± 10.5
Gender		
	Male	3 (6.8)
	Female	41 (93.2)
Weight, kg		66.1 ± 14.1
Occupation		
	Labor	2 (4.5)
	Business	4 (9.1)
	Service industry	5 (11.4)
	Householder	22 (50)
	Teacher	2 (4.5)
	Students	2 (4.5)
	Retired	3 (6.8)
	None	4 (9.1)
Marital status		
	Single	5 (11.4)
	Married	32 (72.7)
	Divorced	3 (6.8)
	Widowed	4 (9.1)
Education		
	Elementary	13 (29.5)
	Junior high	9 (20.5)
	Senior high	5 (11.4)
	Junior college	3 (6.8)
	University	6 (13.6)
	Graduate school	5 (11.4)
	None	3 (6.8)
Income in New Taiwan Dollars (per one month)		
	<$20,000	22 (50)
	$20,000~30,000	10 (22.7)
	$30,000~40,000	4 (9.1)
	$50,000~60,000	3 (6.8)
	$60,000~70,000	2 (4.5)
	$70,000~80,000	1 (2.3)
	≥$80,000	2 (4.5)
Religious beliefs		
	Buddhism	14 (31.8)
	Taoism	18 (40.9)
	Christianity	3 (6.8)
	Yiguan Dao	1 (2.3)
	Other	1 (2.3)
	None	7 (15.9)

**Table 2:** Lymphedema status presenting in the studied patients.

Characteristics	*n* (%)/Mean ± SD
Etiology	
Endometrial cancer	12 (26.0)
Cervical cancer	12 (26.0)
Ovarian cancer	6 (13.0)
Breast cancer	5 (10.8)
Trauma	3 (6.52)
Lymphorrhea	2 (4.34)
Infection	1 (2.17)
Other (Liver donor liver transplantation, Varicose vein)	5 (10.8)
Duration of diagnosis, years	
<1	15 (34.1)
1~<3	9 (20.5)
3~<5	7 (15.9)
5~<10	5 (11.4)
≥10	8 (18.2)
Stage of lymphedema	
I	4 (9.1)
II	4 (9.1)
II,III	34 (77.3)
III	2 (4.5)
Location of lymphedema	
Right leg	23 (52.3)
Left leg	17 (38.6)
Right arm	1 (2.3)
Left arm	3 (6.8)
Limb circumferences, mm	
Pre-op	
Leg	377.8 ± 70.5
Arm	226.2 ± 15.4
Post-op 1 month	
Leg	358.1 ± 43.7
Arm	220 ± 15.9
Post-op 6 months	
Leg	349.8 ± 46.2
Arm	224.8 ± 13.8

**Table 3:** Comparison of lymphedema symptoms among patients preoperatively, one month postoperatively, and six months postoperatively.

Variables	Measured Time	Mean	SD	*p*	Post Hoc
Swelling	1. Pre-op	7.77	2.4	<0.001	3 < 1, 2
	2. Post-op 1 month	7.27	2.07		
	3. Post-op 6 months	3.89	1.82		
Heaviness	1. Pre-op	7.07	3.05	<0.001	3 < 2 < 1
	2. Post-op 1 month	6.07	2.26		
	3. Post-op 6 months	3.34	2.01		
Pain	1. Pre-op	4.27	3.69	<0.001	3 < 2 < 1
	2. Post-op 1 month	2.11	2.26		
	3. Post-op 6 months	0.50	1.00		
Numbness	1. Pre-op	5.82	3.49	<0.001	3 < 2 < 1
	2. Post-op 1 month	4.00	2.00		
	3. Post-op 6 months	2.39	1.73		
Impact on appearance	1. Pre-op	8.18	2.55	<0.001	3 < 1, 2
	2. Post-op 1 month	9.23	1.16		
	3. Post-op 6 months	5.95	1.28		
Joint flexibility	1. Pre-op	6.98	2.40	<0.001	3 < 1, 2
	2. Post-op 1 month	7.61	2.12		
	3. Post-op 6 months	3.98	2.05		
Walking	1. Pre-op	5.43	3.27	<0.001	3 < 1, 2
	2. Post-op 1 month	6.95	2.28		
	3. Post-op 6 months	3.36	2.40		
Climbing stairs	1. Pre-op	5.86	3.31	<0.001	3 < 1, 2
	2. Post-op 1 month	7.00	2.25		
	3. Post-op 6 months	3.30	2.29		
Exercise, dance, play sports	1. Pre-op	7.02	3.07	<0.001	3 < 1, 2
	2. Post-op 1 month	7.27	2.30		
	3. Post-op 6 months	3.80	2.27		
Daily activities	1. Pre-op	5.75	3.45	<0.001	3 < 1, 2
	2. Post-op 1 month	7.41	1.90		
	3. Post-op 6 months	3.16	2.13		
Social activities	1. Pre-op	5.77	3.58	<0.001	3 < 1, 2
	2. Post-op 1 month	7.18	2.18		
	3. Post-op 6 months	3.18	2.04		
Outdoor activities	1. Pre-op	6.25	3.60	<0.001	3 < 1, 2
	2. Post-op 1 month	7.36	1.93		
	3. Post-op 6 months	2.93	1.85		

Evaluation based on a scale of 1 to 10, with 1 representing comfort and 10 extreme discomfort.

**Table 4:** Comparison of quality of life by SF-36 among patients preoperatively, one month postoperatively, and six months postoperatively.

Variables	Measured Time	Mean	SD	*p*	Post Hoc
Physical functioning	1. Pre-op	52.27	25.55	<0.001	3 > 2 > 1
	2. Post-op 1 month	65.68	18.91		
	3. Post-op 6 months	76.13	17.97		
Role-physical	1. Pre-op	32.95	42.03	0.0018	3 > 1, 2
	2. Post-op 1 month	39.20	43.93		
	3. Post-op 6 months	59.65	43.54		
Bodily pain	1. Pre-op	55.83	31.04	<0.001	3, 2 > 1
	2. Post-op 1 month	75.22	21.26		
	3. Post-op 6 months	83.03	18.50		
General health	1. Pre-op	48.84	23.10	<0.001	3, 2 > 1
	2. Post-op 1 month	54.82	21.25		
	3. Post-op 6 months	60.45	20.51		
Vitality	1. Pre-op	54.43	17.95	0.006	3, 2 > 1
	2. Post-op 1 month	60.23	16.95		
	3. Post-op 6 months	61.36	19.30		
Social functioning	1. Pre-op	55.97	27.02	<0.001	3, 2 > 1
	2. Post-op 1 month	68.18	19.15		
	3. Post-op 6 months	72.44	18.00		
Role-emotional	1. Pre-op	36.36	42.42	<0.001	3 > 1, 2
	2. Post-op 1 month	48.48	42.20		
	3. Post-op 6 months	65.15	42.50		
Mental health	1. Pre-op	61.82	19.14	0.680	-
	2. Post-op 1 month	60.73	19.73		
	3. Post-op 6 months	62.55	20.18		
Physical component score	1. Pre-op	47.47	23.95	<0.001	3 > 2 > 1
	2. Post-op 1 month	58.73	18.67		
	3. Post-op 6 months	69.82	19.19		
Mental component score	1. Pre-op	52.14	22.01	<0.001	3 > 2 > 1
	2. Post-op 1 month	59.41	19.59		
	3. Post-op 6 months	65.38	19.53		
Total	1. Pre-op	49.81	22.08	<0.001	3 > 2 > 1
	2. Post-op 1 month	59.07	17.79		
	3. Post-op 6 months	67.60	18.18		

**Table 5:** Comparison of body image using MBSRQ-AS among patients preoperatively, one month postoperatively, and six months postoperatively.

Variables	Measured Time	Mean	SD	*p*	Post Hoc
Appearance evaluation	1. Pre-op	3.12	0.51	0.151	-
	2. Post-op 1 month	3.21	0.57		
	3. Post-op 6 months	3.22	0.58		
Appearance orientation	1. Pre-op	3.28	0.47	<0.001	3, 2 > 1
	2. Post-op 1 month	3.44	0.54		
	3. Post-op 6 months	3.53	0.54		
Body area satisfactions	1. Pre-op	2.90	0.67	0.243	-
	2. Post-op 1 month	2.98	0.55		
	3. Post-op 6 months	3.05	0.55		
Overweight preoccupation	1. Pre-op	2.80	0.70	0.081	-
	2. Post-op 1 month	2.88	0.79		
	3. Post-op 6 months	2.95	0.76		
Self-classified weight	1. Pre-op	3.67	0.63	0.975	-
	2. Post-op 1 month	3.66	0.60		
	3. Post-op 6 months	3.66	0.63		

**Table 6:** Comparison of spiritual health scale among patients preoperatively, one month postoperatively, and six months postoperatively.

Variables	Measured time	Mean	SD	*p*	Post Hoc
Transcendence of adversity	1. Pre-op	4.00	0.61	0.737	-
	2. Post-op 1 month	3.95	0.85		
	3. Post-op 6 months	4.03	0.87		
Interpersonal relationships	1. Pre-op	4.04	0.53	0.056	-
	2. Post-op 1 month	4.21	0.82		
	3. Post-op 6 months	4.20	0.83		
Religious trust	1. Pre-op	3.47	0.77	0.345	-
	2. Post-op 1 month	3.62	1.06		
	3. Post-op 6 months	3.60	1.03		
Meaning of life	1. Pre-op	3.94	0.54	0.240	-
	2. Post-op 1 month	4.10	0.91		
	3. Post-op 6 months	4.05	0.89		
Contemplating nature	1. Pre-op	3.96	0.72	0.268	-
	2. Post-op 1 month	4.14	0.89		
	3. Post-op 6 months	4.06	0.91		
Total	1. Pre-op	3.88	0.52	0.284	-
	2. Post-op 1 month	4.01	0.81		
	3. Post-op 6 months	3.99	0.79		

## Data Availability

The original contributions presented in the study are included in the article, further inquiries can be directed to the corresponding author.
